# Supplementary material for: Nationwide prediction of type 2 diabetes comorbidities
Source: Sci Rep. 2020 Feb 4;10:1776. doi: 10.1038/s41598-020-58601-7 (PMC7000818; doi:10.1038/s41598-020-58601-7)
Supplement: Supplementary file 1 — Dworzynski et al - Supplementary Material. [file 41598_2020_58601_MOESM1_ESM.pdf]

# Supplementary Material

for

*Nationwide prediction of type 2 diabetes comorbidities*

Piotr Dworzynski[1,2], Martin Aasbrenn[1,3], Klaus Rostgaard[2], Mads Melbye[2,4,5], Thomas Alexander Gerds[6], Henrik Hjalgrim[2,+], Tune H Pers[1,2,+]

[1]The Novo Nordisk Foundation Center for Basic Metabolic Research, Faculty of Health and Medical Sciences, University of Copenhagen, Denmark
[2]Department of Epidemiology Research, Statens Serum Institut, Copenhagen, Denmark
[3]Department of Geriatrics and Internal Medicine, Bispebjerg and Frederiksberg Hospital, Copenhagen
[4]Department of Clinical Medicine, University of Copenhagen, Copenhagen, Denmark
[5]Department of Medicine, Stanford University School of Medicine, Stanford, CA, USA
[6]Department of Biostatistics, University of Copenhagen, Copenhagen, Denmark

[+]These authors contributed equally to this work

Correspondence to: tune.pers@sund.ku.dk

**This file includes:**

- Supplementary Note 1: Death as a competing risk
- Supplementary Figures 1 to 9
- Supplementary Tables 1 to 10

### Supplementary Note 1: Death as a competing risk

Individuals who died during the prediction horizon before potentially being diagnosed with the comorbidity were categorized as non-cases. Consequently, death constituted a competing risk to the comorbidity diagnosis. To investigate the extent to which death censoring could impact predictions of the baseline and gradient boosting models we conducted two analyses. First, for each comorbidity and the gradient boosting and the reference model, we investigated five-year incidence of all-cause mortality in each population percentile (in the distribution derived by ranking individuals based on their predicted risk of a given comorbidity). We observed that for all comorbidities incidence of all-cause mortality was generally increasing with the predicted risk of the comorbidity (see Supplementary Fig. 8b for the results for CKD and Supplementary Fig. 9 for the other four comorbidities). Second, we trained and evaluated prediction performance for all-cause mortality using the same procedure as for T2D comorbidities. We found that gradient boosting significantly outperformed all other models (Supplementary Table 10) with AUROCs substantially higher compared to the best AUROCs observed for the T2D comorbidities (AUROC=0.87 vs. HF AUROC=0.80). Furthermore, the gradient boosting feature importances for all-cause mortality, in contrast to the comorbidity prediction feature importances, suggested a more prominent role of hospital diagnoses (among top 7 features three were diagnoses of malignant neoplasms) compared to canonical features and prescriptions (cumulative importances of 32%, 19% and 29%, compared to average cumulative importances of 24.2%, 22.2% and 36.8%, respectively; Supplementary Fig. 8c). These results show that competing risk of death is especially prominent among individuals predicted to be at highest comorbidity risk and that Danish health registers contain information highly predictive of all-cause mortality.

The register-based ML models, due to their high flexibility, could potentially better account for this risk as firstly, the health registers may contain variables, such as diagnoses of terminal diseases e.g. cancers, highly predictive of death. Secondly, more flexible models such as random forest or gradient boosting can better leverage non linear relationships between the features and the outcome by accounting that e.g. very advanced age, otherwise typically positively correlated with a comorbidity risk, may decrease overall comorbidity risk due to increased risk of mortality. In the analogous task of five-year all-cause mortality prediction at first T2D diagnosis, gradient boosting significantly outperformed the reference model achieving a relatively high AUROC of 0.87 when identifying subgroups at high (95th percentile risk ratio of 4.53) risk of death. Furthermore, the best all-cause mortality prediction model was able to identify relatively rare but highly predictive hospital diagnosis features such as cancer diagnoses. Lastly, for HF, CVD, and CKD individuals predicted to be at highest risk of developing a given comorbidity by a register-based model had a lower incidence of all-cause mortality than their counterparts selected by the reference model. This indicates that ML register-based models can outperform the reference model through better estimation of individuals risk of death and weighing it against the risk of a given comorbidity.

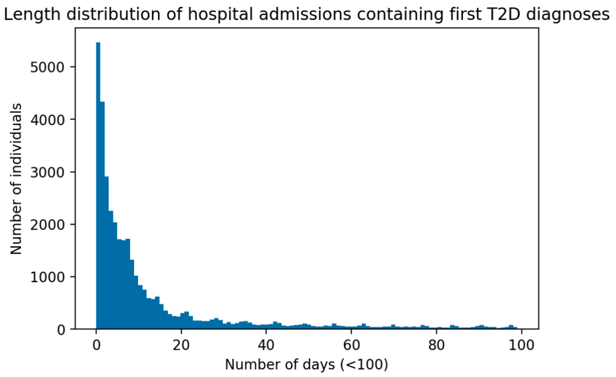


**Supplementary Figure 1.** Histogram of lengths of hospital admissions during which individuals received their first hospital diagnosis of T2D. Individuals with a prior T2D prescription-based diagnosis were excluded. The hospital admissions were limited to those lasting less than 100 days. T2D, type 2 diabetes.

| | **T2D population** | **Chronic kidney disease (BPL 30 days)** | **Chronic kidney disease (BPL 60 days)** |
|---|---|---|---|
| # Individuals | 203,517 | 200,646 | 200,508 |
| # Cases | - | 5,617 (2.80%) | 5,605 (2.80%) |
| # Non-cases | - | 195,029 (97.20%) | 194,903 (97.20%) |
| % Women | 47.03 | 47.18 | 47.18 |
| Median age at T2D diagnosis | 61.44 | 61.32 | 61.31 |
| # Days until outcome | - | 2065.30 | 2089.20 |
| # Features in RFV | - | 6,155 | 6,155 |

**Supplementary Table 1.** Overview of study population characteristics among all newly diagnosed type 2 diabetics (T2D population) and two chronic kidney disease comorbidity populations with buffer period set to 30 and 60 days respectively. RFV, register feature vector; #, number of; BPL, buffer period length.

**Chronic kidney disease (BPL 30 days) (incidence: 0.03)**

| | $AUROC$ | $\Delta AUROC_{RLR}$ | $\Delta AUROC_{LR}$ | $\Delta AUROC_{RF}$ |
|---|---|---|---|---|
| Reference, logistic regression (RLR) | 0.71 (0.69 — 0.73) | | | |
| Logistic regression (LR) | 0.74 (0.72 — 0.76) | 0.04 (0.02 — 0.05) | | |
| Random forest (RF) | 0.74 (0.72 — 0.76) | 0.03 (0.01 — 0.05) | 0.00 (-0.02 — 0.01) | |
| Gradient boosting (GB) | 0.77 (0.76 — 0.79) | 0.07 (0.05 — 0.08) | 0.03 (0.02 — 0.04) | 0.04 (0.02 — 0.05) |

**Chronic kidney disease (BPL 60 days) (incidence: 0.03)**

| | $AUROC$ | $\Delta AUROC_{RLR}$ | $\Delta AUROC_{LR}$ | $\Delta AUROC_{RF}$ |
|---|---|---|---|---|
| Reference, logistic regression (RLR) | 0.70 (0.68 — 0.72) | | | |
| Logistic regression (LR) | 0.74 (0.72 — 0.76) | 0.04 (0.03 — 0.06) | | |
| Random forest (RF) | 0.73 (0.71 — 0.75) | 0.03 (0.01 — 0.05) | -0.01 (-0.03 — 0.01) | |
| Gradient boosting (GB) | 0.77 (0.75 — 0.78) | 0.07 (0.05 — 0.08) | 0.03 (0.02 — 0.04) | 0.04 (0.02 — 0.05) |

**Supplementary Table 2.** Comparison of AUROC measures for each prediction models best parameterization between chronic kidney disease comorbidity populations with buffer period set to 30 and 60 days respectively. We applied a reference- and three register-based models on fifteen years of health register data comprising hospital diagnoses, hospital procedures, drug prescriptions and interactions with primary care contractors to predict five-year risk for chronic kidney disease comorbidity. For each comorbidity, prediction was performed on a T2D population free of that comorbidity at the date of prediction (date of individuals first T2D diagnosis). The reference model was a logistic ridge regression based on canonical features: age, sex, country or region of birth and date of first T2D diagnosis as well as their interactions, while the register-based models were logistic ridge regression, random forest and gradient boosting based on the canonical features as well as hospital diagnoses, hospital procedures, drug prescriptions and interactions with primary care extracted from Danish health registers. Incidences are proportions of cases within comorbidities sub-population at the end of the prediction horizon. Value ranges in brackets represent 95% confidence intervals based on bootstrap sampling. AUROC, area under receiver operating characteristic curve.

| | T2D population | HF | MI |
|---|---|---|---|
| # Individuals | 181,100 | 169,729 | 169,972 |
| # Cases | - | 8,222 (4.84%) | 6,039 (3.55%) |
| # Non-cases | - | 161,507 (95.16%) | 163,933 (96.45%) |
| % Women | 46.98 | 47.53 | 48.13 |
| Median age at T2D diagnosis | 61.71 | 60.95 | 61.24 |
| # Days until outcome | - | 1672.90 | 1546.60 |
| # Features in RFV | - | 6,093 | 6,093 |
| | **ST** | **CVD** | **CKD** |
| # Individuals | 169,870 | 103,971 | 178,586 |
| # Cases | 7,467 (4.40%) | 28,638 (27.54%) | 5,361 (3.00%) |
| # Non-cases | 162,403 (95.60%) | 75,333 (72.46%) | 173,225 (97.00%) |
| % Women | 47.37 | 49.99 | 47.13 |
| Median age at T2D diagnosis | 61.07 | 57.53 | 61.61 |
| # Days until outcome | 1636.50 | 1465.00 | 2060.20 |
| # Features in RFV | 6,093 | 6,093 | 6,093 |

**(a)** one year after first T2D diagnosis

| | T2D population | HF | MI |
|---|---|---|---|
| # Individuals | 165,482 | 154,684 | 154,997 |
| # Cases | - | 8,019 (5.18%) | 5,814 (3.75%) |
| # Non-cases | - | 146,665 (94.82%) | 149,183 (96.25%) |
| % Women | 46.89 | 47.42 | 48.05 |
| Median age at T2D diagnosis | 62.35 | 61.56 | 61.85 |
| # Days until outcome | - | 1659.20 | 1562.80 |
| # Features in RFV | - | 5,929 | 5,929 |
| | **ST** | **CVD** | **CKD** |
| # Individuals | 154,683 | 92,455 | 163,069 |
| # Cases | 7,232 (4.68%) | 26,302 (28.45%) | 5,318 (3.26%) |
| # Non-cases | 147,451 (95.32%) | 66,153 (71.55%) | 157,751 (96.74%) |
| % Women | 47.28 | 49.90 | 47.04 |
| Median age at T2D diagnosis | 61.66 | 58.17 | 62.25 |
| # Days until outcome | 1625.90 | 1452.00 | 2036.50 |
| # Features in RFV | 5,929 | 5,929 | 5,929 |

**(b)** two years after first T2D diagnosis

| | T2D population | HF | MI |
|---|---|---|---|
| # Individuals | 150,228 | 140,053 | 140,418 |
| # Cases | - | 7,667 (5.47%) | 5,492 (3.91%) |
| # Non-cases | - | 132,386 (94.53%) | 134,926 (96.09%) |
| % Women | 46.73 | 47.26 | 47.88 |
| Median age at T2D diagnosis | 62.90 | 62.11 | 62.41 |
| # Days until outcome | - | 1652.20 | 1560.40 |
| # Features in RFV | - | 5,773 | 5,773 |
| | **ST** | **CVD** | **CKD** |
| # Individuals | 139,877 | 81,858 | 147,830 |
| # Cases | 6,896 (4.93%) | 23,993 (29.31%) | 5,178 (3.50%) |
| # Non-cases | 132,981 (95.07%) | 57,865 (70.69%) | 142,652 (96.50%) |
| % Women | 47.18 | 49.69 | 46.91 |
| Median age at T2D diagnosis | 62.22 | 58.77 | 62.78 |
| # Days until outcome | 1592.60 | 1434.60 | 2012.60 |
| # Features in RFV | 5,773 | 5,773 | 5,773 |

**(c)** three years after first T2D diagnosis

| | T2D population | HF | MI |
|---|---|---|---|
| # Individuals | 137,240 | 127,580 | 127,930 |
| # Cases | - | 7,403 (5.80%) | 5,298 (4.14%) |
| # Non-cases | - | 120,177 (94.20%) | 122,632 (95.86%) |
| % Women | 46.38 | 46.89 | 47.51 |
| Median age at T2D diagnosis | 63.56 | 62.75 | 63.07 |
| # Days until outcome | - | 1625.90 | 1543.10 |
| # Features in RFV | - | 5,624 | 5,624 |
| | **ST** | **CVD** | **CKD** |
| # Individuals | 127,240 | 72,742 | 134,874 |
| # Cases | 6,666 (5.24%) | 22,236 (30.57%) | 5,070 (3.76%) |
| # Non-cases | 120,574 (94.76%) | 50,506 (69.43%) | 129,804 (96.24%) |
| % Women | 46.83 | 49.25 | 46.55 |
| Median age at T2D diagnosis | 62.84 | 59.52 | 63.44 |
| # Days until outcome | 1571.40 | 1404.90 | 2018.10 |
| # Features in RFV | 5,624 | 5,624 | 5,624 |

**(d)** four years after first T2D diagnosis

**Supplementary Table 3.** Population characteristics at different dates of prediction. T2D, type 2 diabetes; HF, heart failure; MI, myocardial infarction; ST, stroke; CVD, cardiovascular disease; CKD, chronic kidney disease; RFV, register feature vector; #, number of.

| Logistic regression (LR) | |
|---|---|
| penalty | L2 |
| solver | lbfgs |
| C | 0.6, 0.7, 0.8 |
| max_iter | 300, 400, 500 |

| Random forest (RF) | |
|---|---|
| max_features | sqrt |
| criterion | gini |
| n_estimators | 1000, 1500 |
| max_depth | 12, 14, 16 |

| Gradient boosting (GB) | |
|---|---|
| n_estimators | 200 |
| booster | gbtree |
| eval_metric | auc |
| learning_rate | 0.025, 0.05, 0.1 |
| max_depth | 2, 3, 4, 5 |

**Supplementary Table 4.** Overview of model hyperparameters that were evaluated. For each model type, all combinations of listed hyperparameters were tested to identify those that led to the best average AUROC using 3-fold cross validation on the training set. Parameter names listed correspond to naming in software implementation (Python modules scikit-learn for LR and RF and xgboost for GB). Due to class imbalance (difference between number of cases and non-cases in each population) training error for class representing cases was scaled up by the inverse proportion between cases and non-cases.

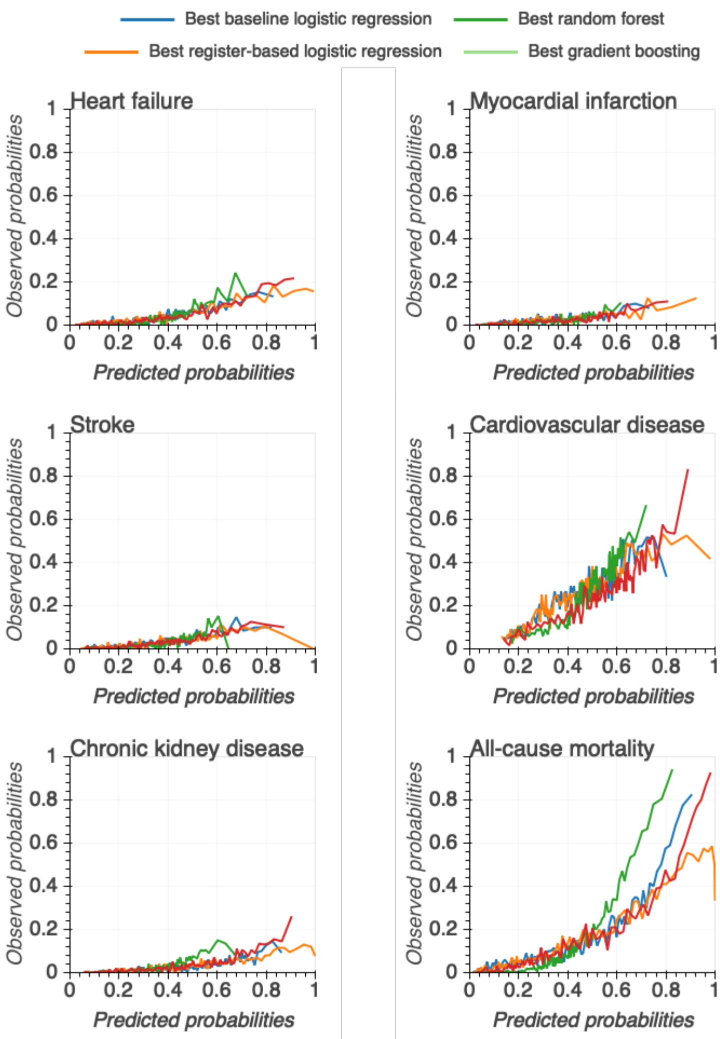


**Supplementary Figure 2.** Validation set calibration curves of uncalibrated best models for each comorbidity and all-cause mortality with date of prediction set to an individual's first type 2 diabetes diagnosis.

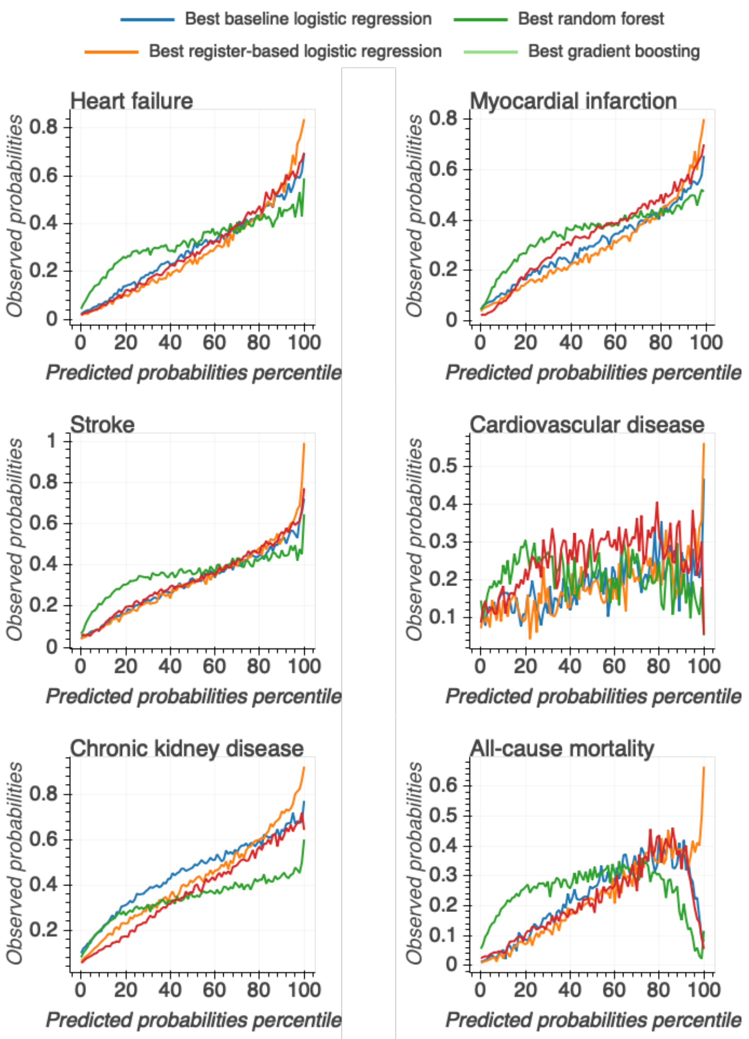


**Supplementary Figure 3.** Validation set calibration error (average difference between observed and predicted outcome probabilities for each predicted probability percentile) of uncalibrated best models for each comorbidity and all-cause mortality with date of prediction set to an individual's first type 2 diabetes diagnosis.

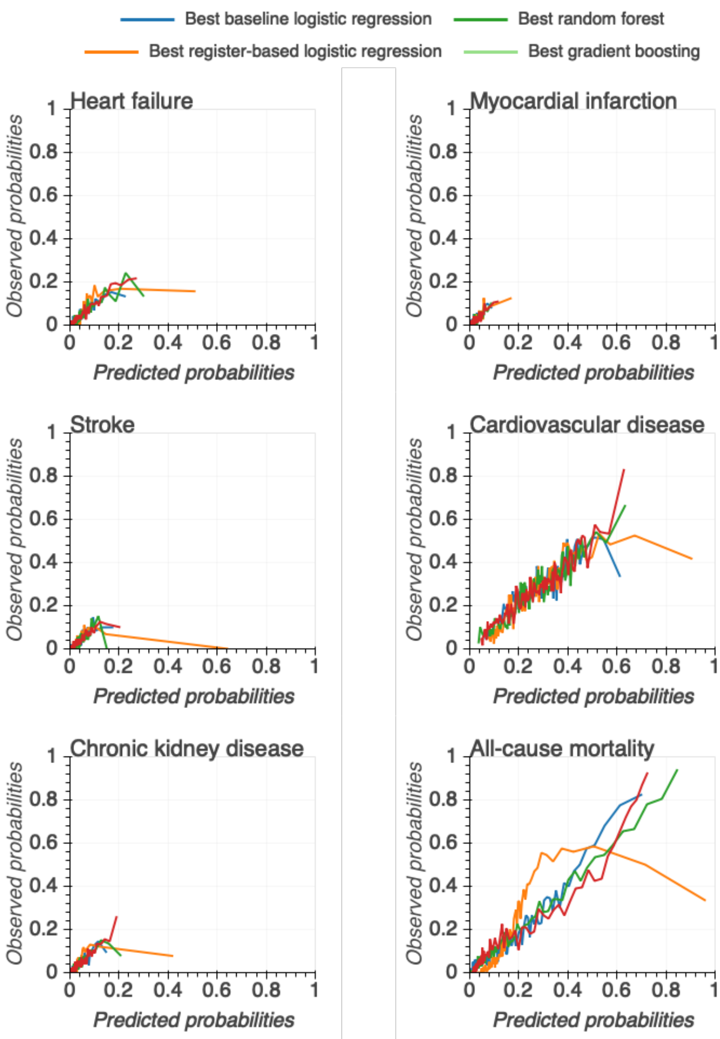


**Supplementary Figure 4.** Validation set calibration curves of calibrated best models for each comorbidity and all-cause mortality with date of prediction set to an individual's first type 2 diabetes diagnosis. Each best model was calibrated using Platt scaling method on the test set.

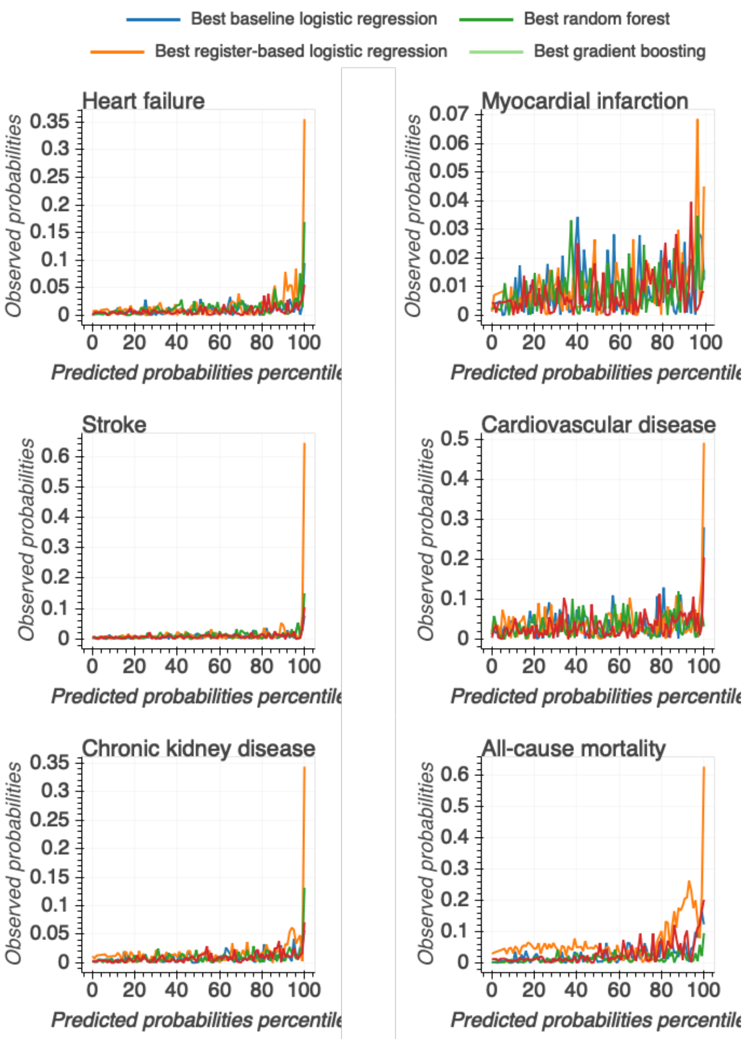


**Supplementary Figure 5.** Validation set calibration error (average difference between observed and predicted outcome probabilities for each predicted probability percentile) of calibrated best models for each comorbidity and all-cause mortality with date of prediction set to an individual's first type 2 diabetes diagnosis. Each best model was calibrated using Platt scaling method on the test set.

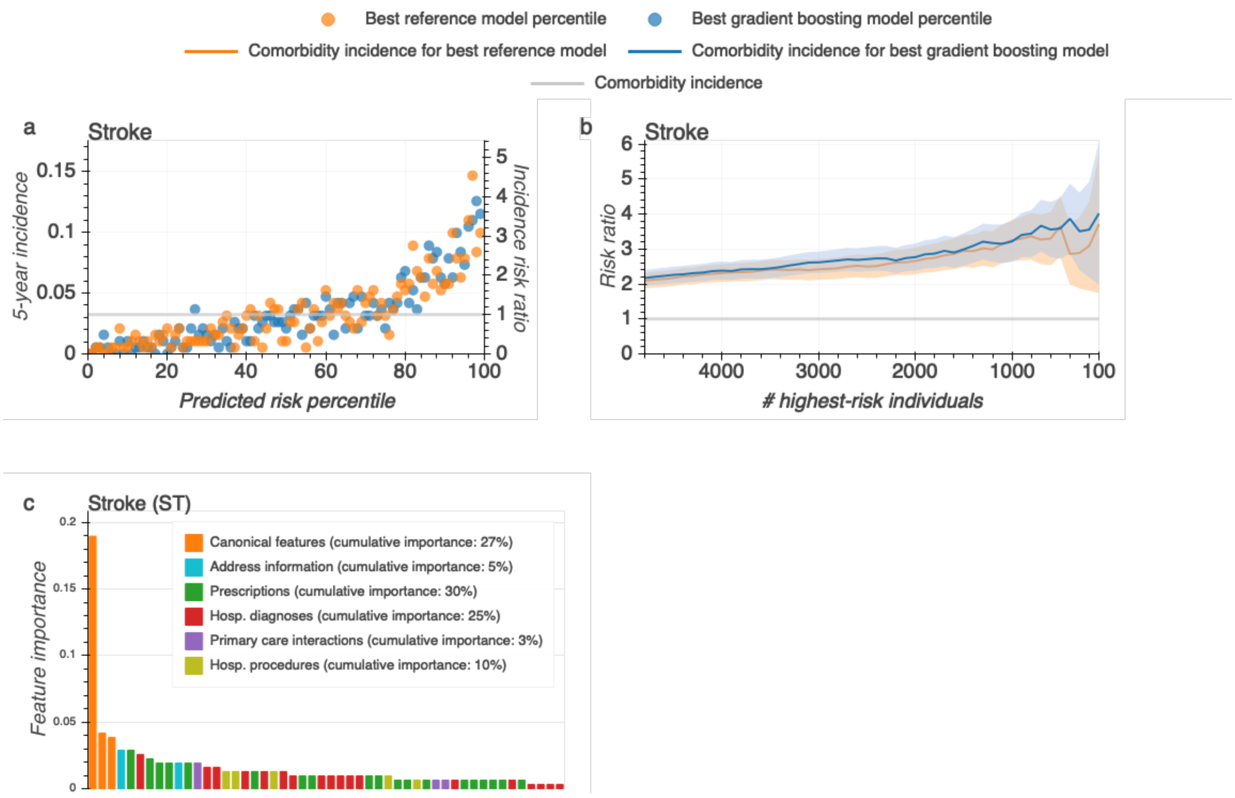


**Supplementary Figure 6.** (**a**) five-year incidence of hospital diagnosis of stroke for population percentiles ranked by risk as predicted by the best gradient boosting (blue) and the best baseline (orange) models. (**b**) Individuals were ranked according to their predicted risk of stroke by the best gradient boosting (blue) and the best baseline (orange) models. For a number of thresholds, shown are risk ratios, calculated as the stroke incidence of individuals ranking above that thresholds over ST incidence in the entire study population. 95% confidence interval (shaded areas) were obtained through bootstrap sampling. (**c**) 50 most predictive features for stroke according to the best gradient boosting models feature importances.

**Heart failure**

| Model | Hyperparameters | AUROC |
|---|---|---|
| GB | max_depth: 2 learning_rate: 0.1 n_estimators: 200 | 0.803 |
| GB | max_depth: 3 learning_rate: 0.05 n_estimators: 200 | 0.803 |
| GB | max_depth: 4 learning_rate: 0.05 n_estimators: 200 | 0.803 |
| GB | max_depth: 3 learning_rate: 0.1 n_estimators: 200 | 0.803 |
| GB | max_depth: 2 learning_rate: 0.05 n_estimators: 200 | 0.802 |
| GB | max_depth: 4 learning_rate: 0.025 n_estimators: 200 | 0.802 |
| GB | max_depth: 4 learning_rate: 0.1 n_estimators: 200 | 0.802 |
| GB | max_depth: 5 learning_rate: 0.025 n_estimators: 200 | 0.801 |
| GB | max_depth: 5 learning_rate: 0.05 n_estimators: 200 | 0.801 |
| GB | max_depth: 3 learning_rate: 0.025 n_estimators: 200 | 0.801 |
| GB | max_depth: 5 learning_rate: 0.1 n_estimators: 200 | 0.800 |
| GB | max_depth: 2 learning_rate: 0.025 n_estimators: 200 | 0.798 |
| RF | n_estimators: 1500 max_depth: 12 | 0.790 |
| RF | n_estimators: 1000 max_depth: 12 | 0.790 |
| RF | n_estimators: 1500 max_depth: 14 | 0.787 |
| RF | n_estimators: 1000 max_depth: 14 | 0.786 |
| LR | C: 0.8 max_iter: 500 | 0.782 |
| LR | C: 0.6 max_iter: 500 | 0.782 |
| LR | C: 0.7 max_iter: 500 | 0.781 |
| RF | n_estimators: 1500 max_depth: 16 | 0.781 |
| RF | n_estimators: 1000 max_depth: 16 | 0.781 |
| LR | C: 0.7 max_iter: 400 | 0.780 |
| LR | C: 0.6 max_iter: 400 | 0.780 |
| LR | C: 0.8 max_iter: 400 | 0.780 |
| LR | C: 0.6 max_iter: 300 | 0.779 |
| LR | C: 0.7 max_iter: 300 | 0.778 |
| LR | C: 0.8 max_iter: 300 | 0.778 |
| RLR | C: 0.6 max_iter: 300 | 0.748 |
| RLR | C: 0.6 max_iter: 400 | 0.748 |
| RLR | C: 0.6 max_iter: 500 | 0.748 |
| RLR | C: 0.7 max_iter: 300 | 0.748 |
| RLR | C: 0.7 max_iter: 400 | 0.748 |
| RLR | C: 0.7 max_iter: 500 | 0.748 |
| RLR | C: 0.8 max_iter: 300 | 0.748 |
| RLR | C: 0.8 max_iter: 400 | 0.748 |
| RLR | C: 0.8 max_iter: 500 | 0.748 |

**Myocardial infarction**

| Model | Hyperparameters | AUROC |
|---|---|---|
| GB | max_depth: 2 learning_rate: 0.1 n_estimators: 200 | 0.730 |
| GB | max_depth: 2 learning_rate: 0.05 n_estimators: 200 | 0.730 |
| GB | max_depth: 3 learning_rate: 0.025 n_estimators: 200 | 0.729 |
| GB | max_depth: 3 learning_rate: 0.1 n_estimators: 200 | 0.729 |
| GB | max_depth: 4 learning_rate: 0.025 n_estimators: 200 | 0.729 |
| GB | max_depth: 3 learning_rate: 0.05 n_estimators: 200 | 0.729 |
| GB | max_depth: 4 learning_rate: 0.05 n_estimators: 200 | 0.728 |
| GB | max_depth: 4 learning_rate: 0.1 n_estimators: 200 | 0.727 |
| GB | max_depth: 2 learning_rate: 0.025 n_estimators: 200 | 0.727 |
| GB | max_depth: 5 learning_rate: 0.025 n_estimators: 200 | 0.727 |
| GB | max_depth: 5 learning_rate: 0.05 n_estimators: 200 | 0.726 |
| GB | max_depth: 5 learning_rate: 0.1 n_estimators: 200 | 0.725 |
| LR | C: 0.6 max_iter: 400 | 0.723 |
| LR | C: 0.8 max_iter: 500 | 0.722 |
| LR | C: 0.6 max_iter: 500 | 0.722 |
| LR | C: 0.7 max_iter: 500 | 0.722 |
| LR | C: 0.7 max_iter: 400 | 0.722 |
| LR | C: 0.8 max_iter: 300 | 0.722 |
| LR | C: 0.8 max_iter: 400 | 0.722 |
| LR | C: 0.6 max_iter: 300 | 0.721 |
| LR | C: 0.7 max_iter: 300 | 0.721 |
| RF | n_estimators: 1500 max_depth: 12 | 0.712 |
| RF | n_estimators: 1000 max_depth: 12 | 0.712 |
| RLR | C: 0.6 max_iter: 300 | 0.703 |
| RLR | C: 0.6 max_iter: 400 | 0.703 |
| RLR | C: 0.6 max_iter: 500 | 0.703 |
| RLR | C: 0.7 max_iter: 300 | 0.703 |
| RLR | C: 0.7 max_iter: 400 | 0.703 |
| RLR | C: 0.7 max_iter: 500 | 0.703 |
| RLR | C: 0.8 max_iter: 300 | 0.703 |
| RLR | C: 0.8 max_iter: 400 | 0.703 |
| RLR | C: 0.8 max_iter: 500 | 0.703 |
| RF | n_estimators: 1000 max_depth: 14 | 0.703 |
| RF | n_estimators: 1500 max_depth: 14 | 0.703 |
| RF | n_estimators: 1000 max_depth: 16 | 0.691 |
| RF | n_estimators: 1500 max_depth: 16 | 0.691 |

**Stroke**

| Model | Hyperparameters | AUROC |
|---|---|---|
| GB | max_depth: 2 learning_rate: 0.1 n_estimators: 200 | 0.734 |
| GB | max_depth: 2 learning_rate: 0.05 n_estimators: 200 | 0.733 |
| GB | max_depth: 3 learning_rate: 0.05 n_estimators: 200 | 0.733 |
| GB | max_depth: 3 learning_rate: 0.1 n_estimators: 200 | 0.733 |
| GB | max_depth: 3 learning_rate: 0.025 n_estimators: 200 | 0.733 |
| GB | max_depth: 4 learning_rate: 0.025 n_estimators: 200 | 0.732 |
| GB | max_depth: 4 learning_rate: 0.05 n_estimators: 200 | 0.731 |
| GB | max_depth: 4 learning_rate: 0.1 n_estimators: 200 | 0.731 |
| GB | max_depth: 2 learning_rate: 0.025 n_estimators: 200 | 0.730 |
| GB | max_depth: 5 learning_rate: 0.025 n_estimators: 200 | 0.730 |
| GB | max_depth: 5 learning_rate: 0.05 n_estimators: 200 | 0.729 |
| GB | max_depth: 5 learning_rate: 0.1 n_estimators: 200 | 0.728 |
| LR | C: 0.8 max_iter: 500 | 0.726 |
| LR | C: 0.6 max_iter: 300 | 0.725 |
| LR | C: 0.8 max_iter: 400 | 0.725 |
| LR | C: 0.6 max_iter: 500 | 0.725 |
| LR | C: 0.6 max_iter: 400 | 0.725 |
| LR | C: 0.8 max_iter: 300 | 0.725 |
| LR | C: 0.7 max_iter: 300 | 0.725 |
| LR | C: 0.7 max_iter: 500 | 0.725 |
| LR | C: 0.7 max_iter: 400 | 0.725 |
| RLR | C: 0.6 max_iter: 300 | 0.717 |
| RLR | C: 0.6 max_iter: 400 | 0.717 |
| RLR | C: 0.6 max_iter: 500 | 0.717 |
| RLR | C: 0.7 max_iter: 300 | 0.717 |
| RLR | C: 0.7 max_iter: 400 | 0.717 |
| RLR | C: 0.7 max_iter: 500 | 0.717 |
| RLR | C: 0.8 max_iter: 300 | 0.717 |
| RLR | C: 0.8 max_iter: 400 | 0.717 |
| RLR | C: 0.8 max_iter: 500 | 0.717 |
| RF | n_estimators: 1500 max_depth: 12 | 0.713 |
| RF | n_estimators: 1000 max_depth: 12 | 0.713 |
| RF | n_estimators: 1500 max_depth: 14 | 0.707 |
| RF | n_estimators: 1000 max_depth: 14 | 0.707 |
| RF | n_estimators: 1500 max_depth: 16 | 0.699 |
| RF | n_estimators: 1000 max_depth: 16 | 0.699 |

**Cardiovascular disease**

| Model | Hyperparameters | AUROC |
|---|---|---|
| GB | max_depth: 4 learning_rate: 0.05 n_estimators: 200 | 0.698 |
| GB | max_depth: 2 learning_rate: 0.1 n_estimators: 200 | 0.698 |
| GB | max_depth: 3 learning_rate: 0.05 n_estimators: 200 | 0.698 |
| GB | max_depth: 3 learning_rate: 0.1 n_estimators: 200 | 0.698 |
| GB | max_depth: 5 learning_rate: 0.05 n_estimators: 200 | 0.698 |
| GB | max_depth: 5 learning_rate: 0.025 n_estimators: 200 | 0.698 |
| GB | max_depth: 4 learning_rate: 0.1 n_estimators: 200 | 0.698 |
| GB | max_depth: 5 learning_rate: 0.1 n_estimators: 200 | 0.697 |
| GB | max_depth: 4 learning_rate: 0.025 n_estimators: 200 | 0.697 |
| GB | max_depth: 2 learning_rate: 0.05 n_estimators: 200 | 0.697 |
| GB | max_depth: 3 learning_rate: 0.025 n_estimators: 200 | 0.695 |
| GB | max_depth: 2 learning_rate: 0.025 n_estimators: 200 | 0.691 |
| RF | class_weight: balanced_subsample n_estimators: 1000 max_depth: 14 | 0.687 |
| RF | class_weight: balanced_subsample n_estimators: 1500 max_depth: 16 | 0.687 |
| RF | class_weight: balanced_subsample n_estimators: 1500 max_depth: 14 | 0.687 |
| RF | class_weight: balanced_subsample n_estimators: 1000 max_depth: 16 | 0.687 |
| RF | class_weight: balanced_subsample n_estimators: 1500 max_depth: 12 | 0.687 |
| RF | class_weight: balanced_subsample n_estimators: 1000 max_depth: 12 | 0.687 |
| LR | class_weight: balanced C: 0.6 max_iter: 400 | 0.682 |
| LR | class_weight: balanced C: 0.7 max_iter: 500 | 0.682 |
| LR | class_weight: balanced C: 0.8 max_iter: 500 | 0.682 |
| LR | class_weight: balanced C: 0.6 max_iter: 500 | 0.682 |
| LR | class_weight: balanced C: 0.7 max_iter: 400 | 0.682 |
| LR | class_weight: balanced C: 0.7 max_iter: 300 | 0.681 |
| LR | class_weight: balanced C: 0.8 max_iter: 400 | 0.681 |
| LR | class_weight: balanced C: 0.8 max_iter: 300 | 0.680 |
| LR | class_weight: balanced C: 0.6 max_iter: 300 | 0.680 |
| RLR | class_weight: balanced C: 0.6 max_iter: 300 | 0.666 |
| RLR | class_weight: balanced C: 0.6 max_iter: 400 | 0.666 |
| RLR | class_weight: balanced C: 0.6 max_iter: 500 | 0.666 |
| RLR | class_weight: balanced C: 0.7 max_iter: 300 | 0.666 |
| RLR | class_weight: balanced C: 0.7 max_iter: 400 | 0.666 |
| RLR | class_weight: balanced C: 0.7 max_iter: 500 | 0.666 |
| RLR | class_weight: balanced C: 0.8 max_iter: 300 | 0.666 |
| RLR | class_weight: balanced C: 0.8 max_iter: 400 | 0.666 |
| RLR | class_weight: balanced C: 0.8 max_iter: 500 | 0.666 |

**Supplementary Table 5.** Area under receiver operating characteristic curve (AUROC) performance for each tested parametrization for heart failure, myocardial infarction, stroke and cardiovascular disease. Date of prediction was set to date of first type 2 diabetes diagnosis. Hyperparameter names correspond to parameter names from the python sklearn package. GB, gradient boosting; RF, random forest; LR, register-based logistic regression; RLR, reference logistic regression.

**Chronic kidney disease**

| Model | Hyperparameters | AUROC |
|---|---|---|
| GB | max_depth: 2 learning_rate: 0.1 n_estimators: 200 | 0.754 |
| GB | max_depth: 3 learning_rate: 0.05 n_estimators: 200 | 0.754 |
| GB | max_depth: 2 learning_rate: 0.05 n_estimators: 200 | 0.753 |
| GB | max_depth: 3 learning_rate: 0.1 n_estimators: 200 | 0.753 |
| GB | max_depth: 3 learning_rate: 0.025 n_estimators: 200 | 0.752 |
| GB | max_depth: 4 learning_rate: 0.05 n_estimators: 200 | 0.752 |
| GB | max_depth: 4 learning_rate: 0.025 n_estimators: 200 | 0.751 |
| GB | max_depth: 2 learning_rate: 0.025 n_estimators: 200 | 0.749 |
| GB | max_depth: 4 learning_rate: 0.1 n_estimators: 200 | 0.749 |
| GB | max_depth: 5 learning_rate: 0.025 n_estimators: 200 | 0.749 |
| GB | max_depth: 5 learning_rate: 0.05 n_estimators: 200 | 0.747 |
| GB | max_depth: 5 learning_rate: 0.1 n_estimators: 200 | 0.745 |
| LR | C: 0.6 max_iter: 500 | 0.730 |
| LR | C: 0.6 max_iter: 400 | 0.729 |
| RF | n_estimators: 1500 max_depth: 12 | 0.729 |
| LR | C: 0.8 max_iter: 400 | 0.729 |
| LR | C: 0.8 max_iter: 500 | 0.729 |
| RF | n_estimators: 1000 max_depth: 12 | 0.729 |
| LR | C: 0.7 max_iter: 500 | 0.727 |
| LR | C: 0.7 max_iter: 400 | 0.727 |
| LR | C: 0.6 max_iter: 300 | 0.726 |
| LR | C: 0.7 max_iter: 300 | 0.726 |
| LR | C: 0.8 max_iter: 300 | 0.724 |
| RF | n_estimators: 1500 max_depth: 14 | 0.718 |
| RF | n_estimators: 1000 max_depth: 14 | 0.717 |
| RF | n_estimators: 1500 max_depth: 16 | 0.701 |
| RF | n_estimators: 1000 max_depth: 16 | 0.700 |
| RLR | C: 0.6 max_iter: 300 | 0.693 |
| RLR | C: 0.6 max_iter: 400 | 0.693 |
| RLR | C: 0.6 max_iter: 500 | 0.693 |
| RLR | C: 0.7 max_iter: 300 | 0.693 |
| RLR | C: 0.7 max_iter: 400 | 0.693 |
| RLR | C: 0.7 max_iter: 500 | 0.693 |
| RLR | C: 0.8 max_iter: 300 | 0.693 |
| RLR | C: 0.8 max_iter: 400 | 0.693 |
| RLR | C: 0.8 max_iter: 500 | 0.693 |

**All-cause mortality**

| Model | Hyperparameters | AUROC |
|---|---|---|
| GB | max_depth: 4 learning_rate: 0.1 n_estimators: 200 | 0.864 |
| GB | max_depth: 5 learning_rate: 0.1 n_estimators: 200 | 0.863 |
| GB | max_depth: 3 learning_rate: 0.1 n_estimators: 200 | 0.863 |
| GB | max_depth: 5 learning_rate: 0.05 n_estimators: 200 | 0.863 |
| GB | max_depth: 4 learning_rate: 0.05 n_estimators: 200 | 0.862 |
| GB | max_depth: 2 learning_rate: 0.1 n_estimators: 200 | 0.861 |
| GB | max_depth: 3 learning_rate: 0.05 n_estimators: 200 | 0.860 |
| GB | max_depth: 5 learning_rate: 0.025 n_estimators: 200 | 0.859 |
| GB | max_depth: 4 learning_rate: 0.025 n_estimators: 200 | 0.857 |
| GB | max_depth: 2 learning_rate: 0.05 n_estimators: 200 | 0.856 |
| GB | max_depth: 3 learning_rate: 0.025 n_estimators: 200 | 0.854 |
| GB | max_depth: 2 learning_rate: 0.025 n_estimators: 200 | 0.847 |
| RF | n_estimators: 1500 max_depth: 16 | 0.845 |
| RF | n_estimators: 1000 max_depth: 16 | 0.845 |
| RF | n_estimators: 1500 max_depth: 14 | 0.842 |
| RF | n_estimators: 1000 max_depth: 14 | 0.842 |
| RF | n_estimators: 1500 max_depth: 12 | 0.838 |
| RF | n_estimators: 1000 max_depth: 12 | 0.838 |
| LR | C: 0.8 max_iter: 500 | 0.827 |
| LR | C: 0.7 max_iter: 500 | 0.827 |
| LR | C: 0.6 max_iter: 500 | 0.826 |
| LR | C: 0.8 max_iter: 400 | 0.826 |
| LR | C: 0.7 max_iter: 400 | 0.826 |
| LR | C: 0.6 max_iter: 400 | 0.825 |
| LR | C: 0.8 max_iter: 300 | 0.823 |
| LR | C: 0.6 max_iter: 300 | 0.822 |
| LR | C: 0.7 max_iter: 300 | 0.822 |
| RLR | C: 0.6 max_iter: 300 | 0.796 |
| RLR | C: 0.6 max_iter: 400 | 0.796 |
| RLR | C: 0.6 max_iter: 500 | 0.796 |
| RLR | C: 0.7 max_iter: 300 | 0.796 |
| RLR | C: 0.7 max_iter: 400 | 0.796 |
| RLR | C: 0.7 max_iter: 500 | 0.796 |
| RLR | C: 0.8 max_iter: 300 | 0.796 |
| RLR | C: 0.8 max_iter: 400 | 0.796 |
| RLR | C: 0.8 max_iter: 500 | 0.796 |

**Supplementary Table 6.** Area under receiver operating characteristic curve (AUROC) performance for each tested parametrization for chronic kidney disease and all cause mortality. Date of prediction was set to date of first type 2 diabetes diagnosis. Hyperparameter names correspond to parameter names from the the python sklearn package. GB, gradient boosting; RF, random forest; RLR, register-based logistic regression; LR, reference logistic regression.

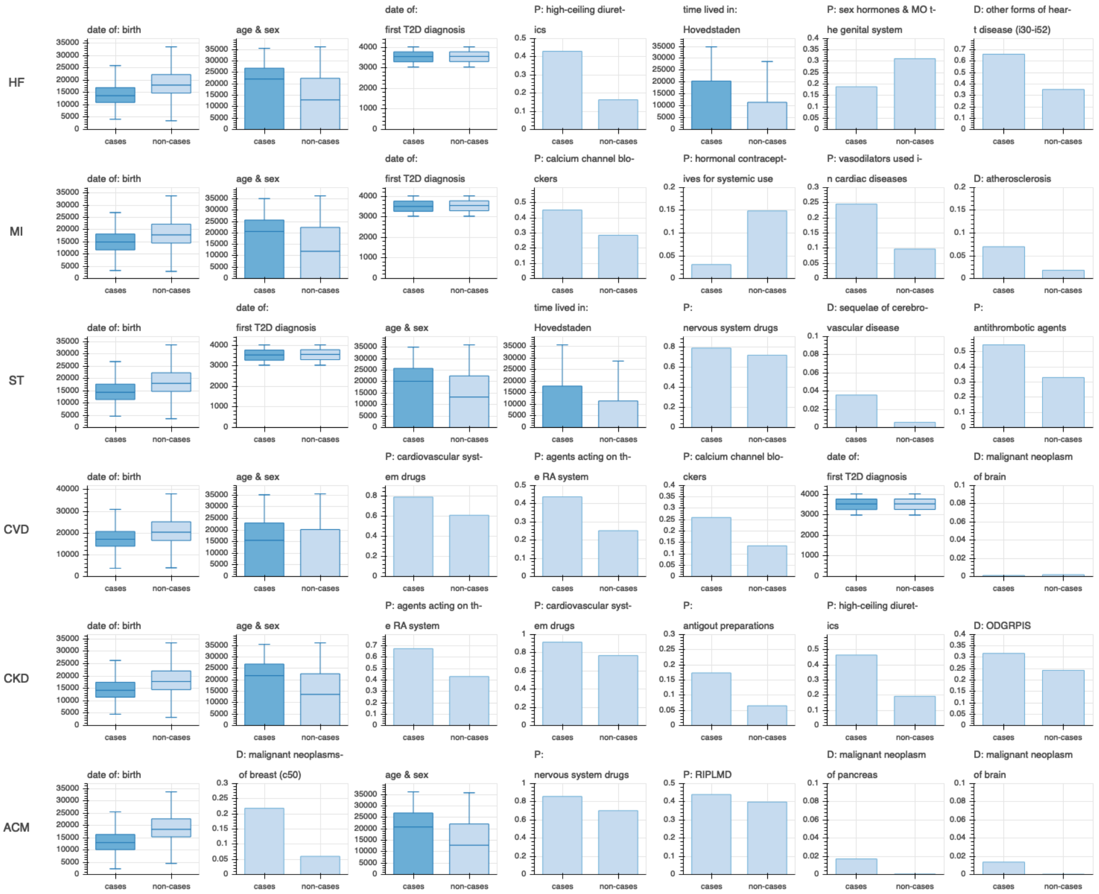


**Supplementary Figure 7.** Top seven most predictive gradient boosting features. For each comorbidity and ACM shown are the top seven features according to gradient boosting feature importance. Feature importance is an estimate of feature's relative contribution to outcome prediction. Box plots show a distribution of a given continuous feature (e.g. age, an interaction between age and sex) among cases and non-cases within validation set. Box plot whiskers represent lowest and highest observations still within 1.5 inter quantile range. To comply with Danish data protection rules, these values as well as values representing 25th, 50th and 75th percentiles were obtained by averaging five closest observations. Bar plots, describing count based features (e.g. count of a given drug prescription, count of a given diagnosis), show the proportion of validation set cases and non-cases with at least a single observation of that feature. HF, heart failure; MI, myocardial infarction; ST, stroke; CVD, cardiovascular disease; CKD, chronic kidney disease; ACM, all-cause mortality; D, diagnosis of; P, prescription of; MO, modulators of; STE, st elevation; RA, renin-angiotensin; ODGRPIS, other disorders of glucose regulation and pancreatic internal secretion; RIPLMD, hmg coa reductase inhibitors and plain lipid modifying drugs.

**Heart failure**

| $t_P$ | $I$ | Gradient Boosting AUROC | Reference AUROC |
|---|---|---|---|
| 0 | 0.04 | 0.80 (0.78 — 0.81) | 0.74 (0.72 — 0.75) |
| 1 | 0.04 | 0.80 (0.78 — 0.81) | 0.74 (0.72 — 0.76) |
| 2 | 0.04 | 0.80 (0.78 — 0.81) | 0.74 (0.72 — 0.76) |
| 3 | 0.04 | 0.81 (0.79 — 0.83) | 0.75 (0.73 — 0.77) |
| 4 | 0.04 | 0.81 (0.79 — 0.82) | 0.74 (0.72 — 0.75) |

**Myocardial infarction**

| $t_P$ | $I$ | Gradient Boosting AUROC | Reference AUROC |
|---|---|---|---|
| 0 | 0.03 | 0.71 (0.69 — 0.73) | 0.68 (0.65 — 0.70) |
| 1 | 0.03 | 0.73 (0.71 — 0.75) | 0.68 (0.66 — 0.71) |
| 2 | 0.03 | 0.74 (0.71 — 0.76) | 0.69 (0.67 — 0.71) |
| 3 | 0.03 | 0.72 (0.70 — 0.75) | 0.69 (0.67 — 0.72) |
| 4 | 0.03 | 0.73 (0.70 — 0.75) | 0.68 (0.66 — 0.71) |

**Stroke**

| $t_P$ | $I$ | Gradient Boosting AUROC | Reference AUROC |
|---|---|---|---|
| 0 | 0.03 | 0.72 (0.70 — 0.74) | 0.71 (0.69 — 0.73) |
| 1 | 0.03 | 0.75 (0.73 — 0.77) | 0.70 (0.68 — 0.72) |
| 2 | 0.03 | 0.75 (0.73 — 0.77) | 0.71 (0.69 — 0.73) |
| 3 | 0.04 | 0.75 (0.73 — 0.77) | 0.72 (0.70 — 0.74) |
| 4 | 0.04 | 0.75 (0.73 — 0.77) | 0.72 (0.70 — 0.74) |

**Cardiovascular disease**

| $t_P$ | $I$ | Gradient Boosting AUROC | Reference AUROC |
|---|---|---|---|
| 0 | 0.26 | 0.69 (0.68 — 0.70) | 0.66 (0.64 — 0.67) |
| 1 | 0.25 | 0.71 (0.70 — 0.73) | 0.67 (0.66 — 0.68) |
| 2 | 0.25 | 0.72 (0.71 — 0.74) | 0.68 (0.67 — 0.70) |
| 3 | 0.25 | 0.73 (0.72 — 0.74) | 0.68 (0.67 — 0.69) |
| 4 | 0.26 | 0.73 (0.72 — 0.74) | 0.69 (0.67 — 0.70) |

**Chronic kidney disease**

| $t_P$ | $I$ | Gradient Boosting AUROC | Reference AUROC |
|---|---|---|---|
| 0 | 0.03 | 0.77 (0.76 — 0.79) | 0.71 (0.69 — 0.73) |
| 1 | 0.03 | 0.77 (0.75 — 0.78) | 0.68 (0.66 — 0.70) |
| 2 | 0.03 | 0.77 (0.75 — 0.79) | 0.70 (0.68 — 0.72) |
| 3 | 0.04 | 0.79 (0.77 — 0.80) | 0.72 (0.70 — 0.74) |
| 4 | 0.04 | 0.79 (0.77 — 0.81) | 0.70 (0.68 — 0.72) |

**All-cause mortality**

| $t_P$ | $I$ | Gradient Boosting AUROC | Reference AUROC |
|---|---|---|---|
| 0 | 0.14 | 0.87 (0.86 — 0.87) | 0.80 (0.79 — 0.81) |
| 1 | 0.14 | 0.87 (0.87 — 0.88) | 0.81 (0.80 — 0.82) |
| 2 | 0.14 | 0.88 (0.87 — 0.88) | 0.81 (0.80 — 0.82) |
| 3 | 0.15 | 0.88 (0.87 — 0.88) | 0.82 (0.81 — 0.83) |
| 4 | 0.16 | 0.87 (0.87 — 0.88) | 0.80 (0.79 — 0.81) |

**Supplementary Table 7.** Prediction performance at one, two, three and four years after individual's first type 2 diabetes diagnosis. $t_P$, time of prediction counted in years since individual's first type 2 diabetes diagnosis; $I$, outcome incidence within study population; AUROC, area under receiver operating characteristic curve which confidence intervals were obtained by bootstrap sampling procedure.

**Heart failure (incidence: 0.04)**

| | AUROC |
|---|---|
| Reference, logistic regression | 0.74 (0.72 — 0.76) |
| Gradient boosting | 0.80 (0.78 — 0.81) |

**Myocardial infarction (incidence: 0.03)**

| | AUROC |
|---|---|
| Reference, logistic regression | 0.68 (0.66 — 0.71) |
| Gradient boosting | 0.73 (0.71 — 0.75) |

**Stroke (incidence: 0.03)**

| | AUROC |
|---|---|
| Reference, logistic regression | 0.70 (0.68 — 0.72) |
| Gradient boosting | 0.75 (0.73 — 0.77) |

**Cardiovascular disease (incidence: 0.24)**

| | AUROC |
|---|---|
| Reference, logistic regression | 0.67 (0.66 — 0.68) |
| Gradient boosting | 0.71 (0.70 — 0.73) |

**Chronic kidney disease (incidence: 0.03)**

| | AUROC |
|---|---|
| Reference, logistic regression | 0.68 (0.66 — 0.70) |
| Gradient boosting | 0.77 (0.75 — 0.78) |

**(a)** one years after first T2D diagnosis

**Heart failure (incidence: 0.04)**

| | AUROC |
|---|---|
| Reference, logistic regression | 0.74 (0.72 — 0.76) |
| Gradient boosting | 0.80 (0.78 — 0.81) |

**Myocardial infarction (incidence: 0.03)**

| | AUROC |
|---|---|
| Reference, logistic regression | 0.69 (0.67 — 0.71) |
| Gradient boosting | 0.74 (0.71 — 0.76) |

**Stroke (incidence: 0.04)**

| | AUROC |
|---|---|
| Reference, logistic regression | 0.71 (0.69 — 0.73) |
| Gradient boosting | 0.75 (0.73 — 0.77) |

**Cardiovascular disease (incidence: 0.25)**

| | AUROC |
|---|---|
| Reference, logistic regression | 0.68 (0.67 — 0.70) |
| Gradient boosting | 0.72 (0.71 — 0.74) |

**Chronic kidney disease (incidence: 0.04)**

| | AUROC |
|---|---|
| Reference, logistic regression | 0.70 (0.68 — 0.72) |
| Gradient boosting | 0.77 (0.75 — 0.79) |

**(b)** two years after first T2D diagnosis

**Heart failure (incidence: 0.04)**

| | AUROC |
|---|---|
| Reference, logistic regression | 0.75 (0.73 — 0.77) |
| Gradient boosting | 0.81 (0.79 — 0.83) |

**Myocardial infarction (incidence: 0.03)**

| | AUROC |
|---|---|
| Reference, logistic regression | 0.69 (0.67 — 0.72) |
| Gradient boosting | 0.72 (0.70 — 0.75) |

**Stroke (incidence: 0.04)**

| | AUROC |
|---|---|
| Reference, logistic regression | 0.72 (0.70 — 0.74) |
| Gradient boosting | 0.75 (0.73 — 0.77) |

**Cardiovascular disease (incidence: 0.25)**

| | AUROC |
|---|---|
| Reference, logistic regression | 0.68 (0.67 — 0.69) |
| Gradient boosting | 0.73 (0.72 — 0.74) |

**Chronic kidney disease (incidence: 0.04)**

| | AUROC |
|---|---|
| Reference, logistic regression | 0.72 (0.70 — 0.74) |
| Gradient boosting | 0.79 (0.77 — 0.80) |

**(c)** three years after first T2D diagnosis

**Heart failure (incidence: 0.04)**

| | AUROC |
|---|---|
| Reference, logistic regression | 0.74 (0.72 — 0.75) |
| Gradient boosting | 0.81 (0.79 — 0.82) |

**Myocardial infarction (incidence: 0.03)**

| | AUROC |
|---|---|
| Reference, logistic regression | 0.68 (0.66 — 0.71) |
| Gradient boosting | 0.73 (0.70 — 0.75) |

**Stroke (incidence: 0.04)**

| | AUROC |
|---|---|
| Reference, logistic regression | 0.72 (0.70 — 0.74) |
| Gradient boosting | 0.75 (0.73 — 0.77) |

**Cardiovascular disease (incidence: 0.25)**

| | AUROC |
|---|---|
| Reference, logistic regression | 0.69 (0.67 — 0.70) |
| Gradient boosting | 0.73 (0.72 — 0.74) |

**Chronic kidney disease (incidence: 0.04)**

| | AUROC |
|---|---|
| Reference, logistic regression | 0.70 (0.68 — 0.72) |
| Gradient boosting | 0.79 (0.77 — 0.81) |

**(d)** four years after first T2D diagnosis

**Supplementary Table 8.** Gradient boosting and baseline model prediction performance for different dates of prediction. Incidence is the proportion of cases within the population. AUROC is area under receiver operating characteristic curve which confidence intervals were obtained by bootstrap sampling procedure. T2D, type 2 diabetes.

**Heart failure ($I_{ts}$: 0.04, $I_{no-ts}$: 0.05)**

| | $AUROC_{ts}$ | $AUROC_{no-ts}$ |
|---|---|---|
| Reference, logistic regression | 0.74 (0.72 — 0.75) | 0.76 (0.74 — 0.77) |
| Logistic regression | 0.77 (0.76 — 0.79) | 0.78 (0.77 — 0.80) |
| Random forest | 0.77 (0.75 — 0.78) | 0.80 (0.79 — 0.81) |
| Gradient boosting | 0.80 (0.78 — 0.81) | 0.81 (0.80 — 0.83) |

**Myocardial infarction ($I_{ts}$: 0.02, $I_{no-ts}$: 0.03)**

| | $AUROC_{ts}$ | $AUROC_{no-ts}$ |
|---|---|---|
| Reference, logistic regression | 0.68 (0.65 — 0.70) | 0.69 (0.67 — 0.71) |
| Logistic regression | 0.70 (0.68 — 0.73) | 0.71 (0.69 — 0.73) |
| Random forest | 0.67 (0.64 — 0.69) | 0.71 (0.69 — 0.73) |
| Gradient boosting | 0.71 (0.69 — 0.73) | 0.72 (0.70 — 0.74) |

**Stroke ($I_{ts}$: 0.03, $I_{no-ts}$: 0.04)**

| | $AUROC_{ts}$ | $AUROC_{no-ts}$ |
|---|---|---|
| Reference, logistic regression | 0.71 (0.69 — 0.73) | 0.72 (0.70 — 0.73) |
| Logistic regression | 0.72 (0.70 — 0.74) | 0.72 (0.70 — 0.74) |
| Random forest | 0.69 (0.67 — 0.71) | 0.71 (0.69 — 0.73) |
| Gradient boosting | 0.72 (0.70 — 0.74) | 0.73 (0.72 — 0.75) |

**Cardiovascular disease ($I_{ts}$: 0.25, $I_{no-ts}$: 0.28)**

| | $AUROC_{ts}$ | $AUROC_{no-ts}$ |
|---|---|---|
| Reference, logistic regression | 0.66 (0.64 — 0.67) | 0.67 (0.66 — 0.68) |
| Logistic regression | 0.68 (0.67 — 0.69) | 0.69 (0.68 — 0.70) |
| Random forest | 0.68 (0.67 — 0.69) | 0.70 (0.69 — 0.71) |
| Gradient boosting | 0.69 (0.68 — 0.70) | 0.71 (0.70 — 0.72) |

**Chronic kidney disease ($I_{ts}$: 0.03, $I_{no-ts}$: 0.03)**

| | $AUROC_{ts}$ | $AUROC_{no-ts}$ |
|---|---|---|
| Reference, logistic regression | 0.71 (0.69 — 0.73) | 0.71 (0.69 — 0.73) |
| Logistic regression | 0.74 (0.72 — 0.76) | 0.75 (0.73 — 0.77) |
| Random forest | 0.74 (0.72 — 0.76) | 0.76 (0.74 — 0.78) |
| Gradient boosting | 0.77 (0.76 — 0.79) | 0.78 (0.76 — 0.80) |

**All-cause mortality ($I_{ts}$: 0.14, $I_{no-ts}$: 0.17)**

| | $AUROC_{ts}$ | $AUROC_{no-ts}$ |
|---|---|---|
| Reference, logistic regression | 0.80 (0.79 — 0.81) | 0.80 (0.79 — 0.81) |
| Logistic regression | 0.83 (0.82 — 0.83) | 0.83 (0.82 — 0.83) |
| Random forest | 0.85 (0.85 — 0.86) | 0.84 (0.84 — 0.85) |
| Gradient boosting | 0.87 (0.86 — 0.87) | 0.86 (0.86 — 0.87) |

**Supplementary Table 9.** Comparison of model performance between time-split and non-time-split models. AUROC measure for each prediction model types best parametrization (according to AUROC measure) for all outcomes compared between time-split ($AUROC_{ts}$; training, test and validation sets were split so that the model is trained on individuals diagnosed with type 2 diabetes historically earlier and evaluated on individuals diagnosed later) and non-time-split model ($AUROC_{no-ts}$; training, test and validation sets were split at random without accounting for date of prediction). $I_{ts}$ and $I_{no-ts}$, outcome incidence within time-split and non-time-split study populations, respectively. AUROC, area under receiver operating characteristic curve which confidence intervals were obtained through bootstrap sampling procedure.

| All-cause mortality (incidence: 0.14) | | | | |
|---|---|---|---|---|
| | $AUROC$ | $\Delta AUROC_{RLR}$ | $\Delta AUROC_{LR}$ | $\Delta AUROC_{RF}$ |
| Reference, logistic regression (RLR) | 0.80 (0.79 — 0.81) | | | |
| Logistic regression (LR) | 0.83 (0.82 — 0.83) | 0.03 (0.02 — 0.03) | | |
| Random forest (RF) | 0.85 (0.85 — 0.86) | 0.05 (0.05 — 0.06) | 0.03 (0.02 — 0.03) | |
| Gradient boosting (GB) | 0.87 (0.86 — 0.87) | 0.07 (0.06 — 0.07) | 0.04 (0.04 — 0.05) | 0.01 (0.01 — 0.02) |

**Supplementary Table 10.** Area under receiver operating characteristic curve for prediction of all-cause mortality by the best reference and register-based models. Compared to the prediction of T2D comorbidities, all models achieved relatively high AUROCs, with all register-based models outperforming the reference model and gradient boosting outperforming the other register-based models. T2D, type 2 diabetes; AUROC, area under receiver operating characteristic curve.

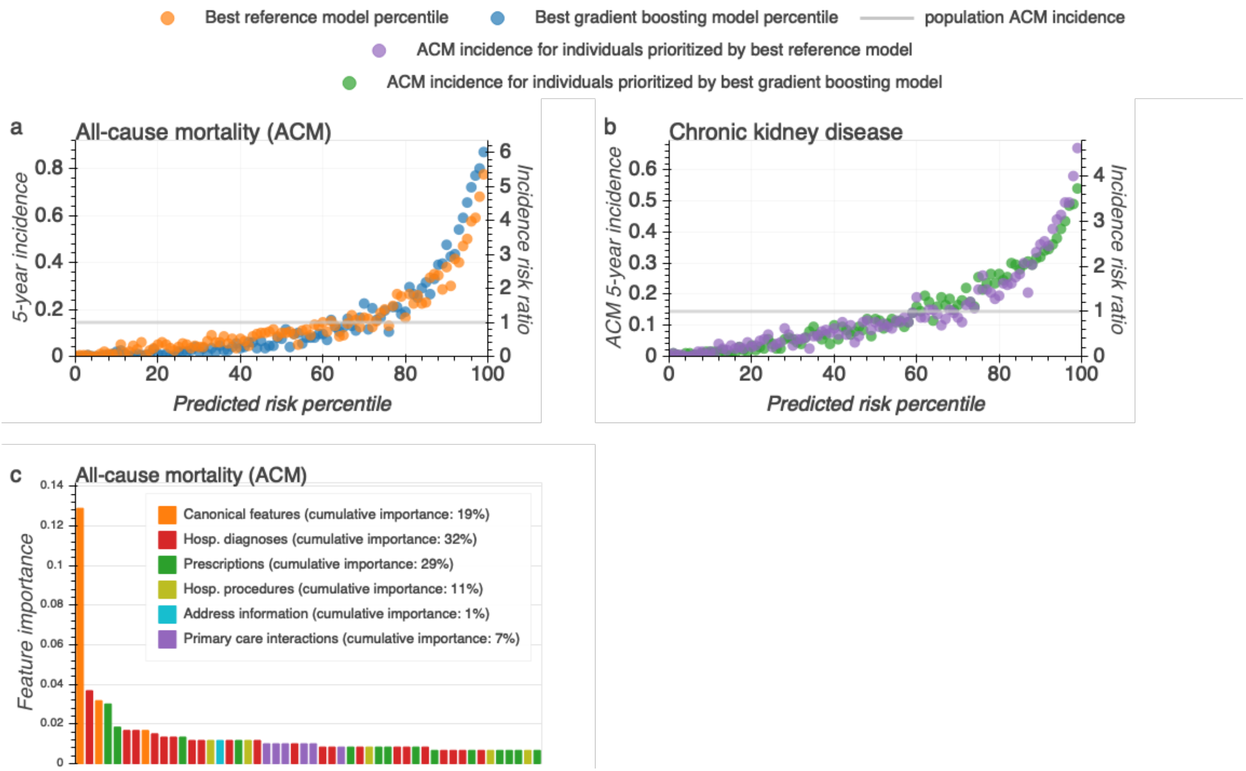


**Supplementary Figure 8.** Death is a competing risk for diagnosing T2D comorbidities and its effect may be estimated by ML models implicitly. We investigated whether health register data and machine learning model could predict five-year risk of all-cause mortality (ACM) using the same procedure as for T2D comorbidities. (**a**) five-year incidence of ACM for population percentiles ranked by ACM risk predicted by gradient boosting (blue) and reference (orange) models. At the 95th percentile, both models identified individuals ranking multiples above the overall population incidence (ACM risk ratio of 4.53, and 3.46, respectively). (**b**) Incidence of ACM among population percentiles according to predicted risk of chronic kidney disease. Individuals stratified by the register-based model had a similar or lower risk of ACM than those binned into top percentiles by the reference model. (**c**) 50 register features most predictive of ACM according to the gradient boosting models feature importances. Unlike the case of the investigated T2D comorbidities, hospital diagnoses features were the most important followed by prescriptions second and canonical features third.

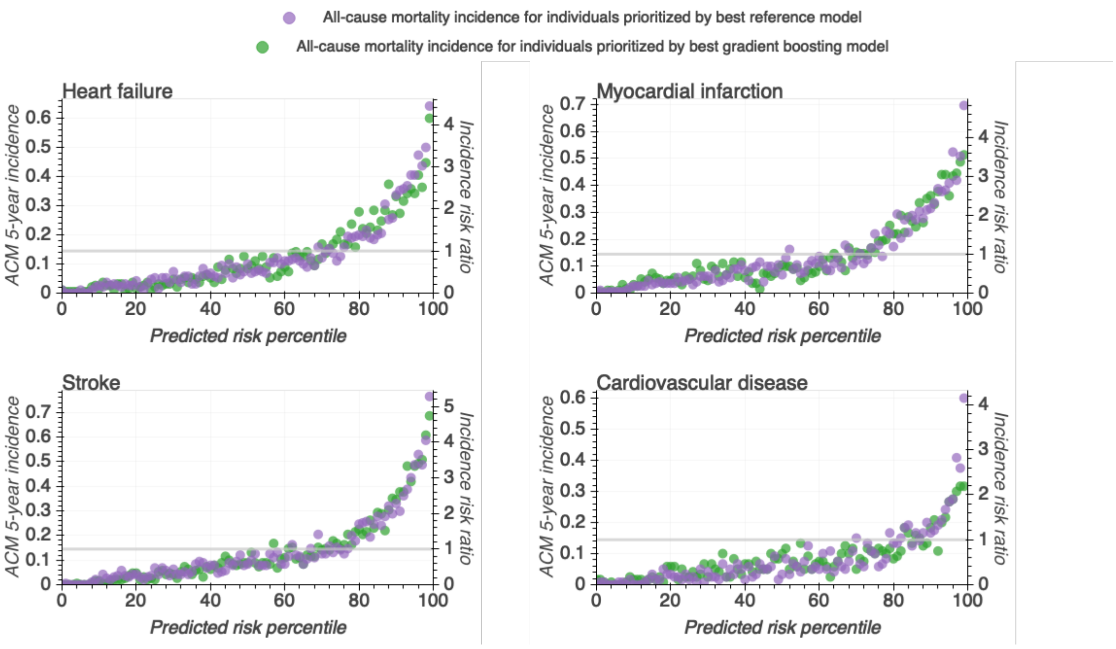


**Supplementary Figure 9.** All-cause mortality five-year incidence in population percentiles ranked by predicted type 2 diabetes comorbidity risk by the best gradient boosting (green) and best baseline (violet) models.
